# Supplementary material for: No island-effect on glucocorticoid levels for a rodent from a near-shore archipelago
Source: PeerJ. 2020 Feb 18;8:e8590. doi: 10.7717/peerj.8590 (PMC7034373; doi:10.7717/peerj.8590)
Supplement: Table S1 — Numbers apply to the following for each trapping session: Tn –total trap nights, Trip –total disturbed traps, Ind–individually tagged white-footed mice caught, and Capt –total white-footed mice captures. [file peerj-08-8590-s001.docx]

**Table S1. Summary of trapping success during each trapping session for white-footed mice (*Peromyscus leucopus*) in Thousand Islands National Park, Canada.**

|  | **Site** | **Island area (ha)** | **Distance to mainland (m)** | **Summer 2015** | | | | **Spring 2016** | | | | **Summer 2016** | | | |
| --- | --- | --- | --- | --- | --- | --- | --- | --- | --- | --- | --- | --- | --- | --- | --- |
|  |  |  |  | **Tn** | **Trip** | **Ind** | **Capt** | **Tn** | **Trip** | **Ind** | **Capt** | **Tn** | **Trip** | **Ind** | **Capt** |
| **Islands** | Aubrey | 6.8 | 755.5 | 289 | 112 | 2 | 6 | 147 | 93 | 0 | 0 | 98 | 68 | 0 | 0 |
|  | Beaurivage | 4.9 | 348.8 | - | - | - | - | 50 | 16 | 4 | 4 | - | - | - | - |
|  | Camelot | 9.4 | 3636.2 | 147 | 82 | 2 | 2 | - | - | - | - | - | - | - | - |
|  | Constance | 3.4 | 462.6 | 50 | 25 | 12 | 12 | 70 | 40 | 3 | 4 | 96 | 41 | 8 | 9 |
|  | Georgina | 10.1 | 191.1 | 123 | 4 | 6 | 10 | 98 | 11 | 2 | 4 | 98 | 78 | 0 | 0 |
|  | Grenadier | 554.3 | 1037.9 | 100 | 8 | 27 | 38 | 98 | 20 | 7 | 9 | 98 | 5 | 15 | 18 |
|  | Hill | 555.9 | 438.1 | 98 | 3 | 20 | 20 | 147 | 72 | 5 | 6 | 98 | 23 | 12 | 12 |
|  | Lindsay | 14.4 | 429.1 | 98 | 6 | 36 | 52 | 98 | 12 | 9 | 14 | 98 | 12 | 25 | 36 |
|  | Mermaid | 1.7 | 1045.7 | - | - | - | - | 20 | 98 | 4 | 4 | - | 57 | - | - |
|  | McDonald | 17.4 | 512.2 | 98 | 32 | 28 | 38 | 147 | 0 | 3 | 4 | 98 | - | 15 | 17 |
|  | Thwartway | 40.1 | 2837.3 | 147 | 23 | 22 | 28 | 98 | 54 | 7 | 10 | 98 | 73 | 10 | 10 |
|  |  |  |  |  |  |  |  |  |  |  |  |  |  |  |  |
|  | *Subtotal* |  |  | 1150 | 295 | 155 | 206 | 973 | 416 | 44 | 59 | 782 | 357 | 85 | 102 |
|  |  |  |  |  |  |  |  |  |  |  |  |  |  |  |  |
| **Mainland** | Escot Property | |  | 98 | 1 | 14 | 27 | 98 | 10 | 17 | 21 | 98 | 19 | 14 | 22 |
|  | Jones Creek 1 | |  | 120 | 15 | 25 | 30 | 147 | 41 | 6 | 10 | 98 | 38 | 13 | 23 |
|  | Jones Creek 2 | |  | - | - | - | - | - | - | - | - | 98 | 13 | 9 | 12 |
|  | Landon Bay | |  | 196 | 23 | 9 | 16 | 147 | 102 | 3 | 3 | 147 | 64 | 7 | 9 |
|  | Mallorytown | |  | - | - | - | - | 98 | 16 | 12 | 18 | 98 | 7 | 12 | 17 |
|  |  |  |  |  |  |  |  |  |  |  |  |  |  |  |  |
|  | *Subtotal* |  |  | 414 | 39 | 48 | 73 | 490 | 169 | 38 | 52 | 539 | 141 | 55 | 83 |
|  |  |  |  |  |  |  |  |  |  |  |  |  |  |  |  |
|  | *Total* |  |  | 1564 | 334 | 203 | 279 | 1463 | 585 | 82 | 111 | 1321 | 498 | 140 | 185 |

Numbers apply to the following for each trapping session: **Tn** – total trap nights, **Trip** – total disturbed traps, **Ind** – individually tagged white-footed mice caught, and **Capt** – total white-footed mice captures.
