# Supplementary material for: No island-effect on glucocorticoid levels for a rodent from a near-shore archipelago
Source: PeerJ. 2020 Feb 18;8:e8590. doi: 10.7717/peerj.8590 (PMC7034373; doi:10.7717/peerj.8590)
Supplement: Table S2 [file peerj-08-8590-s002.docx]

Table S2. Sampling dates (dates that traps were checked) for island and mainland sites in Thousand Islands National Park during three trapping periods.

|  | **Site** | **Summer 2015** | **Spring 2016** | **Summer 2016** |
| --- | --- | --- | --- | --- |
| **Islands** | Aubrey | August 1, 22-24, 30-31 | June 8, 10, 11 | August 12-13 |
|  | Beaurivage | - | June 10-11 | - |
|  | Camelot | August 16-18 | - | - |
|  | Constance | July 10-11 | June 16-18 | August 18-19 |
|  | Georgina | July 9-11 | June 16-17 | August 18-19 |
|  | Grenadier | July 3-4 | June 14-15 | August 23-25 |
|  | Hill | August 14-15 | May 26-27,31 | August 2-3 |
|  | Lindsay | July 31-August 1 | May 23-25 | July 29-30 |
|  | McDonald | July 16-17 | May 22-23 | July 27-28 |
|  | Mermaid | - | June 10-11 | - |
|  | Thwartway | July 18, 29-30 | June 1,4 | August 8-9 |
|  |  |  |  |  |
| **Mainland** | Escot Property | July 19-20 | June 5-6 | August 20-21 |
|  | Jones Creek 1 | July 7-8 | May 17-19 | July 22-23 |
|  | Jones Creek 2 | - | - | July 22-23 |
|  | Landon Bay | August 25-28 | May 19-20, 31 | July 24-26 |
|  | Mallorytown | - | June 5-6 | August 4-5 |
