# Supplementary figures and images for: No island-effect on glucocorticoid levels for a rodent from a near-shore archipelago
Source: PeerJ. 2020 Feb 18;8:e8590. doi: 10.7717/peerj.8590 (PMC7034373; doi:10.7717/peerj.8590)

### Wild Mouse Hair Parallelism

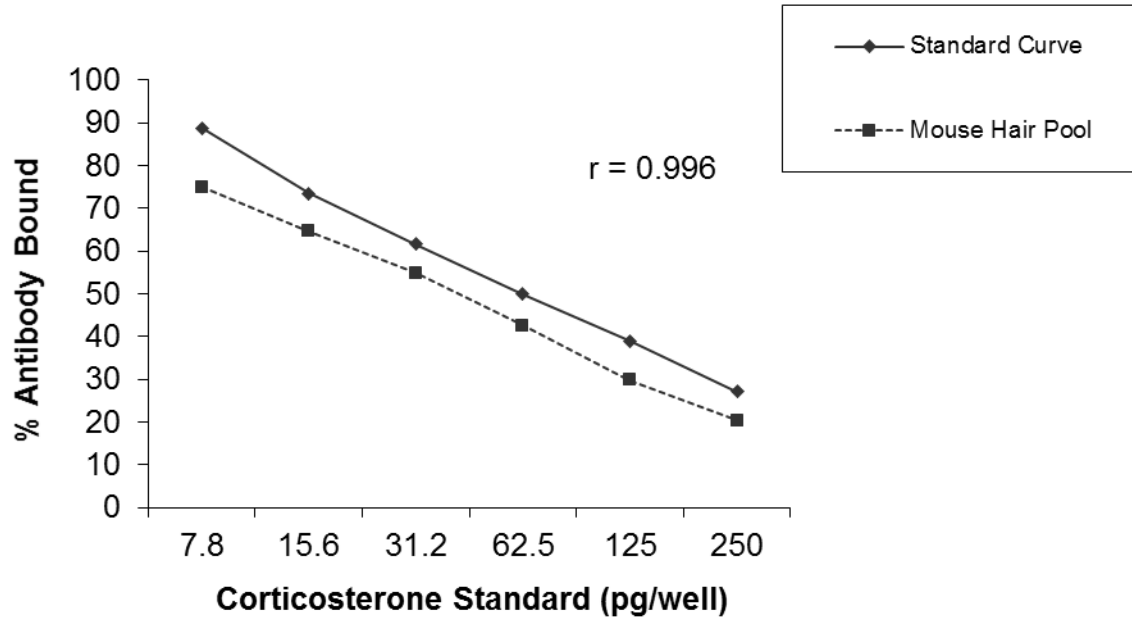

### Wild Mouse Fecal Parallelism

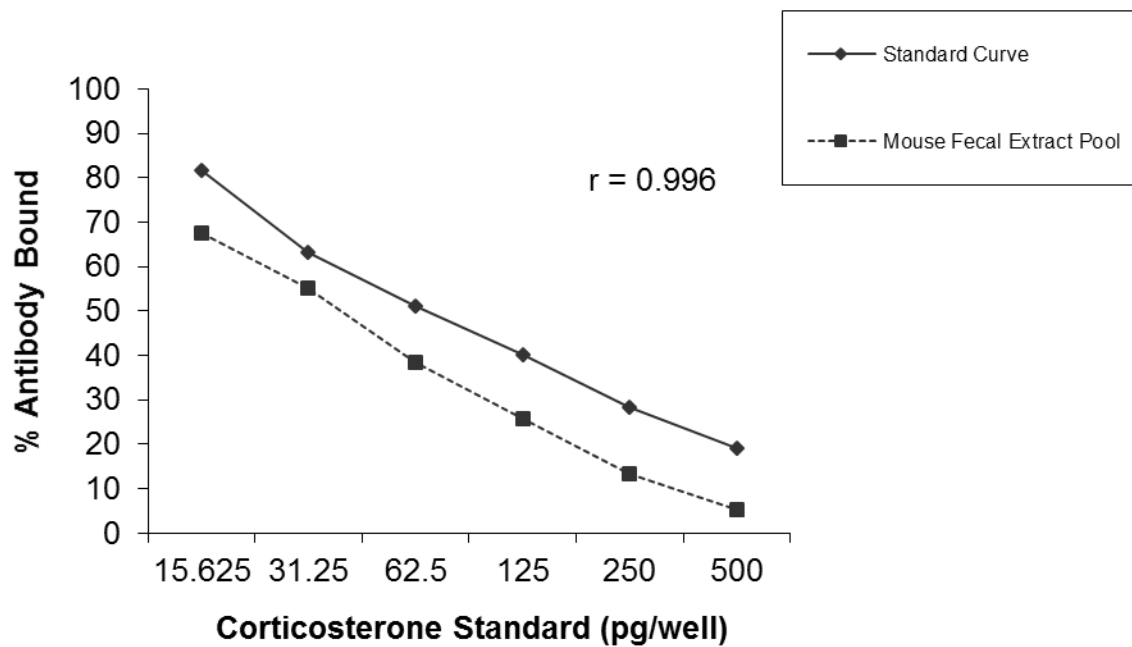

Supplement: Figure S1 [file peerj-08-8590-s003.pdf]
