# Supplementary material for: No island-effect on glucocorticoid levels for a rodent from a near-shore archipelago
Source: PeerJ. 2020 Feb 18;8:e8590. doi: 10.7717/peerj.8590 (PMC7034373; doi:10.7717/peerj.8590)
Supplement: Data S2 [file peerj-08-8590-s005.rtf]

Column Name	Units	Description	
season	-	Spring (May-June) or summer (July-August)	
type	-	Habitat type where individuals were captured: island or mainland	
CPUE_Corrected	Individuals per trap night	Catch-per-unit-effort calculated for each site during each trapping session and corrected for number of tripped traps 	
island_size	Hectares	Area calculated for each island: measured using ArcGIS	
dist	Metres	Shortest distance between each island and the mainland: measured using ArcGIS	
site	-	Specific trapping site where each individual was captured	
date	-	Date on which each individual was sampled	
year	-	Year of capture: either 2015 or 2016	
species	-	Pl – Peromyscus leucopus	
weight_g	Grams	Body mass for each individual measured using a spring-scale	
sex	-	Sex determined for each individual	
reproductive_status	-	Reproductive status determined for each individual: pregnant or NA	
maturity_code	-	Maturity code determined for each individual: juvenile, subadult or adult	
tag_number	-	Tag number applied to each individual 	
hair_cort_ng_g	ng/g	Hair corticosterone concentration for samples from each individual	
fecal_cort_ng_g	ng/g	Fecal corticosterone metabolites concentration for samples from each individual	
